# Classification of users’ transportation modalities from mobiles in real operating conditions

**DOI:** 10.1007/s11042-021-10993-y

**Published:** 2021-05-26

**Authors:** Claudio Badii, Angelo Difino, Paolo Nesi, Irene Paoli, Michela Paolucci

**Affiliations:** Department of Information Engineering, Distributed Systems and Internet Tech lab, DISIT Lab, University of Florence, Florence, Italy

**Keywords:** User behaviour analysis, Smart city, Mobile phones, Transportation modes, Classification model, Machine learning

## Abstract

The modern mobile phones and the complete digitalization of the public and private transport networks have allowed to access useful information to understand the user’s mean of transportation. This enables a plethora of old and new applications in the fields of sustainable mobility, smart transportation, assistance, and e-health. The precise understanding of the travel means is at the basis of the development of a large range of applications. In this paper, a number of metrics has been identified to understand whether an individual on the move is stationary, walking, on a motorized private or public transport, with the aim of delivering to city users personalized assistance messages for: sustainable mobility, health, and/or for a better and enjoyable life, etc. Differently from the state-of-the-art solutions, the proposed approach has been designed to provide results, and thus collect metrics, in *real operating conditions* (imposed on the mobile phones as: a range of different mobile phone kinds, operating system constraints managing Applications, active battery consumption manager, etc.). The paper reports the whole experimentations and results. The solution has been developed in the context of Sii-Mobility Km4City Research Project infrastructure and tools, performed with the collaboration of public transport operators, and GDPR compliant. The same solution has been used in Snap4City mobile Apps with experiments performed in Antwerp and Helsinki.

## Introduction

With the complete digitalization of the public and private transportation networks, the capability of understanding the users’ behaviour and the mean of transportation have become important. The presence of GPS, accelerometers, sensors on mobile phones has made possible to create solutions exploiting the users’ behaviour and context. Information from GPS and accelerometers available on mobile phones can be combined with contextual information regarding city and mobility and transport information to assess and predict user behaviour. The new smart city applications are based on the fact that the city users are producing data, and thus they are contributing to the city as a cyber physical system. City users are at the same time information providers and recipients of personalized suggestions and information [18]. The understanding of user behaviour is the first step to provide contextualized suggestions and assistance to people on the move via mobile phones in smart city, putting city users in the loop. For example, to allow the city users to receive suggestions to take more virtuous behaviour, consume less energy, making more sustainable their transportation, having a healthier life walking more, saving money parking closer, remaining in certain areas (for the COVID-19 directives), finally to monitor their reaction and acceptance level. The research addressed in this article aims to understand the users’ mean of traveling taking into account contextual data and data coming from the mobile phones. The correct classification of transportation means can be also used for providing suggestions in the context of public or private transportation. For example, it may have sense to suggest getting down the bus at the next stop to walk a bit, or suggest parking the car in different place, respectively, using public transportation instead of the private one. Thus, the above-described problem is reconducted to the classification problem of the transportation modality/mean (car, bus, walk, bike, etc.), exploiting real time data coming from the mobile phone devices and contextual information. Please note that, the contextual data are strongly different in different part of the city, and also change over time, for example busses have different timeline and paths: so that users are moving in the real space.

As described in the following section of related works, the problem of understanding the travel mean of users has been many times addressed, but not working in real operating conditions. An early review of transportation mode detection techniques has been proposed in [7].

Most of them, assume data collected from the mobile phones with high rates and high precision, identifying models only taking data in strongly controlled conditions: such as limited number of mobile phone types, limited number of users directly engaged to keep the mobile App running in foreground, and/or on specific orientation, etc. This means that those solutions have not addressed real operating conditions. In the presence of real conditions, (i) data are sporadic, (ii) the data rate is low and not constant, (iii) the quality of data is not uniform since sensors of different mobile phones have different precision and response, (iv) operating system may push the mobile App in background and may apply energy saving rules to App. These are just examples of the complexity of understanding the mean of transportation in *real operating conditions*. This also means that both data and methods must be completely re-elaborated to work on real conditions.

### Related works

The problem of classifying users’ mean of travelling has been addressed by a number of approaches in different research areas [22, 23]: *Location Based Services* (LBS), *Transportation Services* (TS) and *Human Geography* (HG). The LBS solutions aim to understand the transportation meaning in real-time to provide useful information to the user whenever he/she asks. In TSc approaches, the correct segmentation of a trajectory is privileged with respect to velocity of response: [8]. The HG approaches focus on the segmentation of a trajectory into parts with domain-specific semantics: it is common to first split trajectories into segments where the object is stationary or moving. In LBS, the transportation means’ classification is regarded as an online process: an algorithm that provides the current transportation mode of the user in real-time or quasi real-time. To this end, different types of data/sensors have been exploited: GPS, accelerometer and their combination. In [25], Stenneth et al. compared five different models using data collected from GPS classifying the users’ travelling means in six categories (walk, train, driving, stationary, bus, bike). In [16], Prelipcean et al. proposed a study that involves only accelerometer data. They have obtained an 84.9% precision and an 85.3% recall for seven transportation modes, by using both AdaBoost and Decision Tree (two-stages classification). In [31], Yu et al. have compared three different classifiers (Decision Tree obtaining 84.81% average accuracy, AdaBoost 87.16% average accuracy, and SVMs with 90.66% average accuracy). In [29], Wang et al. have considered a small dataset of 12 h (5544 samples of six transportation modes) from 7 different users, obtaining a 70% accuracy with a Decision Tree algorithm. In [24], Reddy et al. demonstrated that, taking into account of both GPS and accelerometer the accuracy can be improved. They have achieved a 93.6% accuracy using a combination of Decision Tree and Hidden Markov Model (two-stages classification), with both accelerometer and GPS features involved, using a sampling rate made a distinction among different type of non-motorized motion (walking, running, biking), vehicular and random movements, using the accelerometer sensors of mobile. In [19], Manzoni et al. trained a Decision Tree classification model obtaining an 82.14% accuracy (with a GPS and accelerometer sampling frequency rate of 1 s and 0.04 s respectively). In [1], Ashqar et al. proposed a two-layer hierarchical classifier to predict five classes of transportation mode (car, bus, walk, run, bike), achieving a 97% accuracy. In [30], Yanyun et al. presented a Convolutional Neural Networks (CNN) based method to automatically extracting features for the identification of transportation means, thus achieving 98,6% accuracy to distinguish between train, bus, car, metro.

In Table 1, a summary of the state-of-the-art solutions for understanding the travel means is reported (the table report also additional experiments/papers with respect to those commented above).

**Table 1:** Related Work implementation overview. (*) the value reported refers to Precision %

Authors	Classes	Data exploited	Sampling	#users	#features	# of mobile phone types	Accuracy %
Wang et al. [29]	Stationary, Walk, Bike, Bus, Car, Metro	Accel	0.03 s (accel)	7	23	1	70
Manzoni et al. [19]	Stationary, Walk, Bike, Motorcycle, Car, Bus, Metro, Train	Gps Accel	1 s (gps) 0.04 s (accel)	4	1	1	82.1
Reddy et al. [24]	Stationary, Walk, Run, Bike, Vehicle	Gps Accel	1 s (gps) 0.03 s (accel)	16	4	1	93.6
Stenneth et [25]	Stationary, Walk, Bike, Car, Bus, Train	Gps Gis	14 s (gps)	6	7	3	93.4
Hemminki [16]	Stationary, Walk, Car, Bus, Train, Metro, Tram	Accel	0.01 s (accel)	16	27 + 5	3	84.9 (*)
Prelipcean [23]	Walk, Bike, Car, Bus, Metro, Train, Ferry	Gps Accel	50 m (gps) 0.2 s (accel)	9	11	–	90.8 (*)
Yu et al. [31]	Stationary, Walk, Run, Bike, Vehicle (Motorcycle, Car, Bus, Metro, Rail, Train)	Accel	0.03 s (accel)	224	22 + 8	1	90.6
Yanyun et al. [30]	Train, Metro, Bus, Car	Accel	0.01 s (accel)	30	169	1	98,6
Ashqar et al. [1]	Car, Bus, Bike, Run, Walk	Gps Accel	0.04 s (gps)0.01 s (accel	10	80	2	97,0

As it can be noted from Table 1, almost all the state-of-the-art solutions adopted very *high rates for GPS data acquisition, with limited number of mobile phones and type***s**. So that, those solution are almost unfeasible in *real operating conditions*. Mobile phone operating systems allow to keep the high rates (in the order of seconds) only when applications are running in foreground. In most cases, the precisions provided has been obtained with limited set of mobile phones types/devices in unrealistic conditions.

### Research aims and article organization

The aim of our research has been to realize a solution overcoming the state-of-the-art solutions to classify the transportation modes to deliver personalized services for:

- sustainable mobility, to incentivize ecological transportation choices, suggesting alternative public mean of transportation (bus/tram) instead of the private car/motorbike;
- healthy suggestions, better and enjoyable life, to stimulate users in dedicating a part of their time and moving needs to exercising their body. For example, suggesting getting out of the bus in advance, park in other locations, etc.;
- implementing city strategies to change city user attitudes [3, 5].

With this purpose, the real-time identification of a private transportation mode (car or motorbike) has a central role in assistance messages delivery. Therefore, according to the above described real operating conditions, the techniques have to produce high classifications accuracy to identify user transportation mean, in the presence of (i) large discontinuities samples of data (from sensors and sporadic communications to the central computation modules), (ii) relevant differences which may be due to the different kind of mobile phone features in terms of sensors and precision, (iii) real operative conditions despite the power safe procedures in the mobile phones. Therefore, the proposed solution overcomes the above-mentioned solutions at the state of the art, working on real conditions. The solution has been validated on a real application (delivered to the users via official App stores such as Google Play Store, Apple App Store, and accepted by common users, see “*Tuscany where what….*” on the stores). As described in the following, it is capable to cope with (i) constrains introduced by mobile phone manufactures on battery usage for background and foreground services, (ii) no restrictions on the usage of mobile phone (e.g., orientation, foreground or background), (iii) no limitation on using specific mobile phone types.

The paper is focused on the classification of the transportation mode/means whether an individual is: (i) stationary, (ii) walking, (iii) on a motorized private transport (car or motorbike), or (iv) in a public transport (tram, bus or train). To this aim a large experimentation of the solution has been conducted, with the support of Public Transport Operators: ATAF, BUSITALIA and CTTNORD. The classification model proposed has been produced by using open and real-time data of Sii-Mobility/Km4City project and infrastructure (which is national smart city project of Italian Ministry of Research for terrestrial mobility and transport, http://www.sii-mobility.org). Sii-Mobility is based on Km4City data aggregation and analytics infrastructure (https://www.km4city.org) in the Tuscany area, Italy, and its Smart City solutions, recently integrated into https://www.snap4city.org.

This paper has been organized as described in the following. Section 2 reports the general architecture for data collection, from mobile phones to server, and related data analytics. It also includes a description of the applicative scenario. Section 3 describes the classification methods adopted to identify and validate the predictive models and framework. In Section 4, a list of the identified metrics is reported, mainly related to: baseline, GPS, accelerometer, and historical data. Section 5 proposes a comparison of predictive models exploiting the collected data from Km4City, to arrive at identifying the best resulting approach in terms of classification precision and recall. It also presents a comparison between a one-step classification method (a single multi-class classifier) and a hierarchical approach. Section 5. F describes the results in the context of the most relevant applicative scenario tested. Conclusions are drawn in Section 6.

## Architecture and data collection overview

**Fig. 1:**
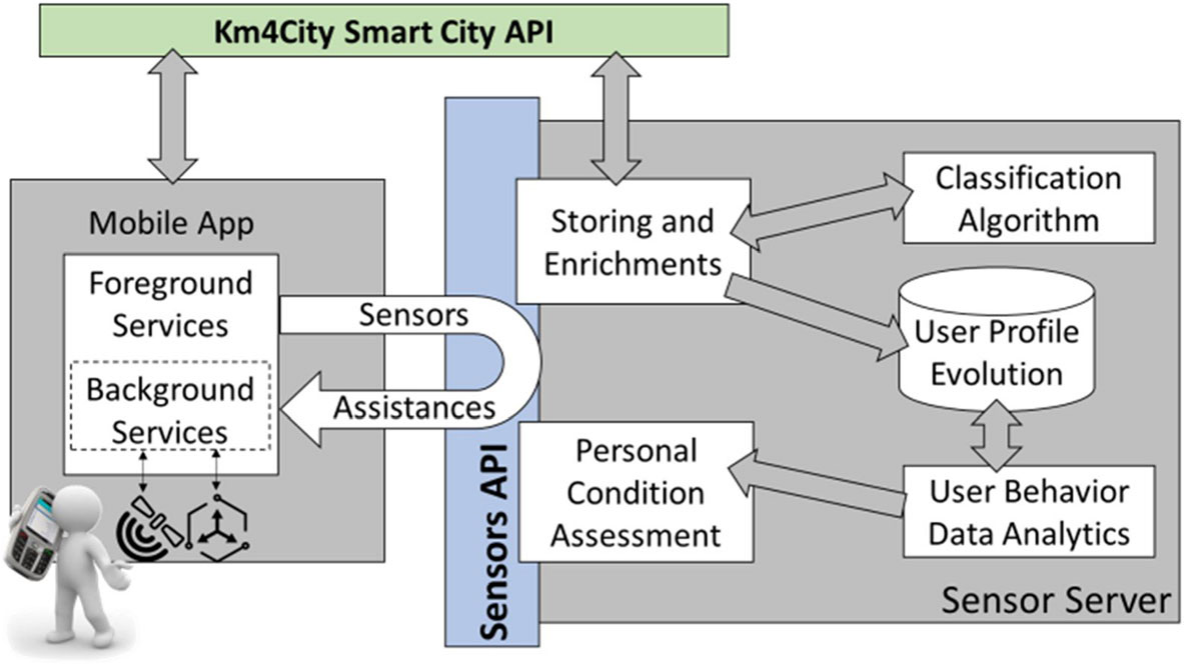
System architecture

The proposed solution relies on a client-server architecture, where the mobile applications can be installed on different operating systems and different mobile phones [4]. The sensors’ values collected on the mobile phone (client-side) are sent to the server that enriches them with additional contextual information (by taking into account the GIS, geographical information system and knowledge), etc., as described in the sequel. At the same time, the server executes the real time classification algorithm to compute the transportation mean for each user. The information is stored on server as the current user’s travel means, and to form the history of conditions. On this basis, strategies are triggered when the user behaviour reaches certain specific conditions – i.e., to assist and/or engage the users in daily activities (even rewarding them, in the cases of virtuous behaviour). For example, a strategy for stimulating the city users may be based on a firing condition to send a suggestion to city users that use the private car to perform the same trip path at least 3 times per week, and at the same time the trip could be easily performed by using a public transportation. Thus, the system may inform those city users of the possible alternatives, and some of them may follow the suggestion. As a result, by performing the user behaviour analysis in real time, the solution may detect the acceptance of the suggestion by detecting of change of behaviour and may automatically reward the user with a bonus or discount, and deliver congratulations. See [3], on rules and strategies. Figure 1 provides a high-level overview of the software architecture and its main components.

The **Mobile App** may execute services/processes in background or just in foreground depending on the operating systems. Therefore, different data collection strategies are used on different mobile phones and operating systems. Android mobile phones accept to have background processes; thus, the data collection service may work in background remaining always/periodically active. In certain operating systems, services running in background are not allowed; therefore, data can be only collected when the App is in foreground. Both data collection services (foreground and background) follow the same operations independently on the implementation languages: Java for the background service on Android and JavaScript for other operating systems, since the Mobile App has been developed in Apache Cordova.

The data collected on the mobile applications is called by us as “*Sensor Data Package*” (considering the mobile phone as a sensor), and includes: (1) position and movement of the user’s mobile phone through sensors’ and derived information such as GPS latitude and longitude, speed and acceleration; (2) mobile phone characteristics such as: operating system type, application version, mobile phone model, statistics on the mobile application usage, and some details on configuration; and (3) the user profile characteristics (language, category (tourist, commuter, student, etc.)). This information is used to contextualize and personalize the user experience. The collection of *Sensor Data Package* allows to collect a GPS position with a minimum refresh time of 30s, which dynamically managed by the operating system power safe strategy. Moreover, some mobile phones and operating systems derive a sort of GPS position from the network connection (cellular connection and/or Wi-Fi hotspots locations), or by using some fused/mixed strategies, which combine the different sources. This approach may produce a location even when the GPS location is not available (for example in buildings). For the above reason, our solution is keeping trace of the *Location Measure kind* which can be GPS, Network (cellular position or Wi-Fi) or mixt. If, at least one location mode is active, two different cases may occur: (1) it was not possible to get e fresh GPS location of the mobile phone at that time (for example, the user is inside a building where the GPS receiver fails to get information from satellites) or (2) the current location is not up to date (for example, the position was obtained by the mobile phone before entering in a building, and remain the last valid position for long time, getting old. In both these cases, a position update is needed, and if it is not possible, a simply ALIVE message is sent to the server, communicating the other values of the *Sensor Data Package*, they include the accelerometers information which can be collected anyway from the mobile phone.

The *Sensor Data Package* is collected in a buffer and sent by the mobile phone to the server periodically by the active background or foreground services. When the sending of the last packages is successful, the data sent are deleted from mobile phone buffer.

**Fig. 2:**
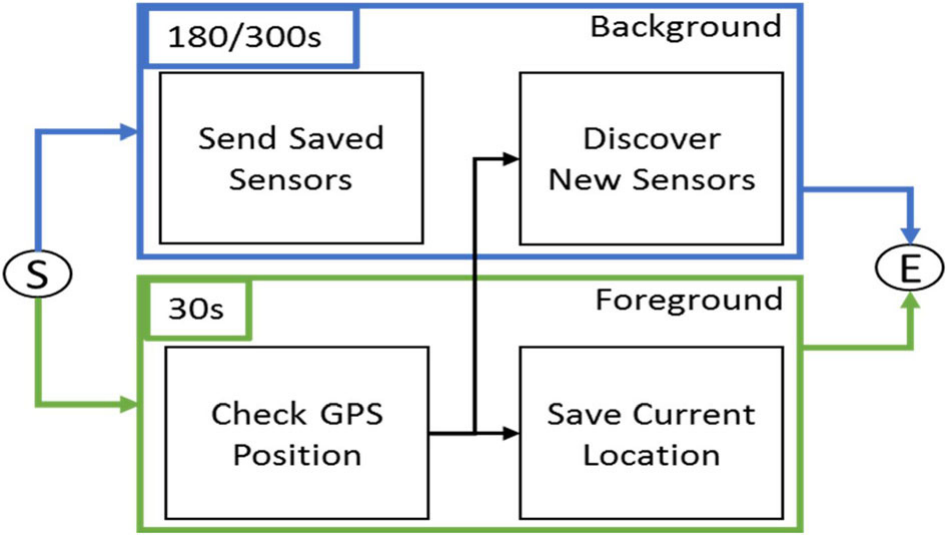
Data recovery service timing

In Fig. 2, the protocol’s operations performed to collect and send data to the server are shown. The block called “*Save Current Location*” stores (every time the GPS position is available) a list of data used to calculate a number of features of the mobile phone movements at that time. Both background and foreground services perform the same operations in their corresponding operative conditions of the App. The *server-side process* (see Fig. 1) collects all the data sent by the mobile applications and computes several features for enriching each single record of data (package) associated with a given timestamp of measure. Among the computed features we have: the distance/proximity of the GPS coordinates of the mobile phone with respect to the railway, or bus lines, or highway, cycling path, and/or parking zones. This kind of geographical information is retrieved from the Km4City knowledge base in Big Data technology by using the Smart City API [2, 32], interface to semantically integrated information for App and services [6]. Other computed features are the average velocity of the last period, etc., as described in the sequel.

Finally, the process on the server puts in execution the classification algorithm to compute the user’s transportation mean. This information is also stored point by point for further processing, higher level reasoning. And thus, to produce contextual messages to be delivered to the user’s mobile phone according to the strategies identified by the municipality or by some city operator which are coded by rules [3]. Please note that, the classification approach of transportation mean has to predict the traveling means for the next time slot, in order to provide suggestions in time and not only with the delay due to the data collection – sending – and computing process.

### Applicative scenario

**Fig. 3:**
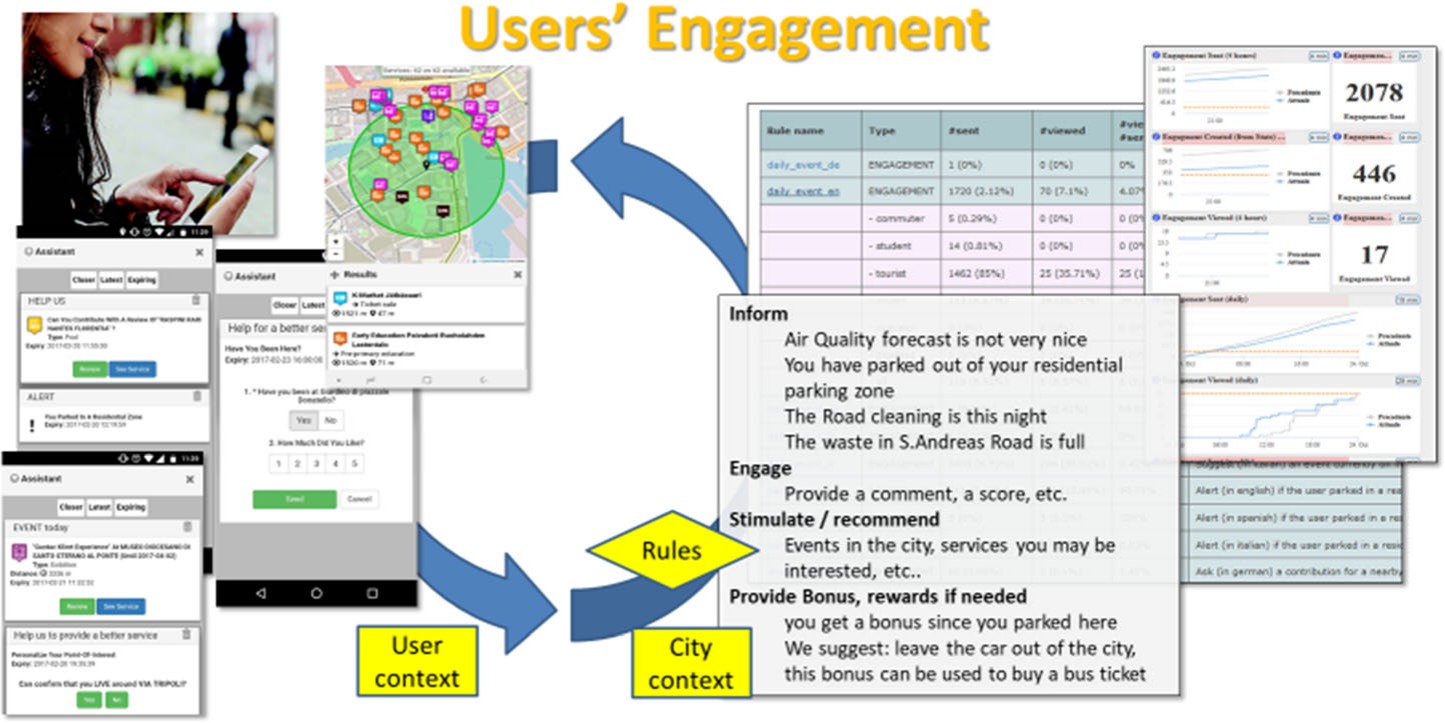
User Engagement scenario

The proposed solution has been used during a campaign carried out in the period April–July 2018 to produce contextual stimulus to the citizens of three metropolitan areas in Tuscany (Firenze, Prato, Pisa) via a Mobile Application. The aim has been to engage users to stimulate virtuous behaviours on the basis of different strategies (e.g., serious game) defined and promoted by city operators. The solution needed to extract the citizen’s behaviour as transport means and context information as location semantics: work, home, school, etc. Thus, on the basis of the context assessed in real time, the solution can provide stimuli via engagement’s messages, also collecting feedbacks on the service and on the city infrastructure; and finally, inform and reward the users that accepted the suggestions (see Fig. 3). All results need to be reported in a scoreboard highlighting the best user in terms of user’s participation and follow ups to perform statistical analysis. The solution has to be flexible enough to allow the creation of contextualized custom rules to send and manage the engagements dialogue. For example, some of them can reward to stimulate a change or an action by the users, while others can stimulate and provide the rewards only when the user has performed the action and the solution recognizes its occurrence [3].

## Classification techniques compared

This section presents an overview of techniques considered to create a solution for classifying the transportation mean of the users in the move. During the experiments, several unsatisfactory techniques have been tested, thus, among the possible techniques, we have chosen to present here only the comparison of those that produce the best results, namely: *Random Forests, Extremely Randomized Trees, Extreme Gradient Boosting, Super Learner and Hierarchical approach***.** The approaches adopted in the literature such as: SVM, Decision Tree, and AdaBoost have not produce satisfactory results with respect to the above mentioned and reported solutions. Random Forest can be regarded as a generalization of Decision Tree. The AdaBoost belongs to the same family of the more powerful Extreme Gradient Boosting approach.

The usage of Classification Trees approaches, also called ensemble methods, i.e., *Extremely Gradient Boosting* and *Random Forests* methods, have potential advantages in the predictive model’s construction. Please note that Classification Trees are machine-learning methods which are used for constructing exploration, description, and prediction models. *Classification Trees* are free of distributional assumptions and they can handle different types of responses, such as categorical, numeric, multivariate, censored and dissimilarity matrices; they are invariant to predictors monotonic transformations; the presence of missing values in the predictors are handled with a minimal loss of information. On the basis of the above described properties, the adoption of classification and regression trees -- i.e., Extremely Randomized Trees or Random Forest methods which are free of distributional assumptions – potentially provide an advantage for the construction of predictive models.

As further step, the multi-class problem has been divided in a collection of binary classification problems: they were analysed using the *Super Learner* algorithm, combining the different learning techniques above. Moreover, a *Hierarchical* approach has been proposed and compared with the above approaches based on a single multi-class classifier.

**Fig. 4:**
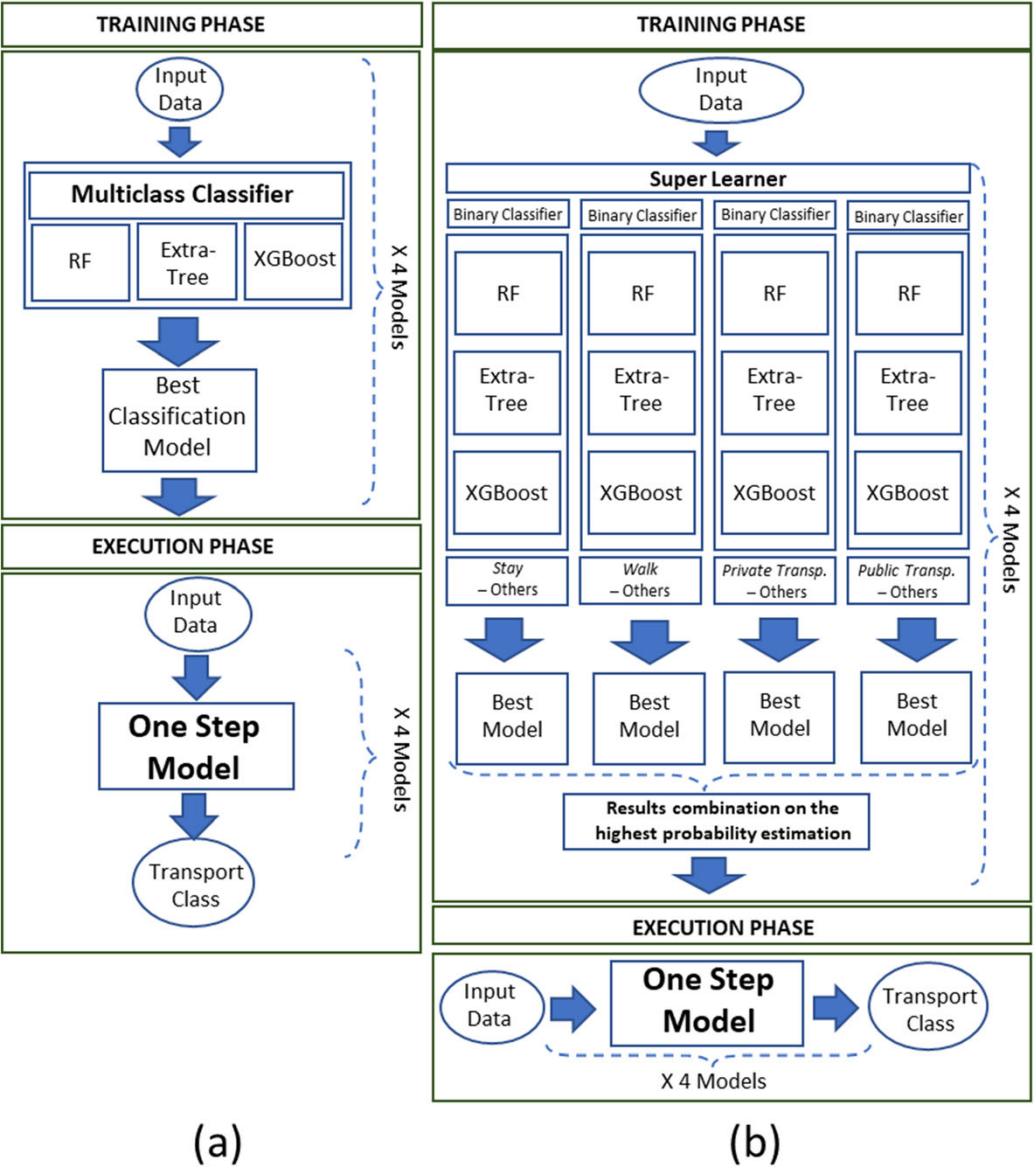
Comparison of One Step models: **a** classic and **b** Super Learner

Therefore, for completeness, a short overview of the above-mentioned approaches is reported in the next subsections. In Fig. 4, a schema of the processes adopted is reported for both classic classifiers (a) and Super Learner (b).

### Random forests

*Random Forests* algorithm has been proposed by Breiman [9] as an improvement of the Tree Bagging approach. In Random Forests, a different bootstrap sample from the original data was used to construct each tree. For each tree of the collection, each split is determined by a randomly chosen subset of predictors; this procedure reduces the correlation between predictors of the individual trees, and each tree has the same expectation.

### Extreme gradient boosting

Gradient Boosting [13] is a way to reduce the variance, respect to other decision tree methods. The Boosting Trees algorithm is an evolution of the boosting methods application. Boosting methods [12] performs classifications by weighted majority vote, and they have the advantage to fit many trees of different dimensions to reweighted versions of the training data.

In *Gradient Boosting*, small regression/classification trees are built sequentially from the gradient of the previous tree loss function (pseudo-residuals), and in order to produce an incremental improvement in the model, at each iteration, trees are built from random sub-samples of the dataset.

Basically, given a modality *i* with a vector of covariates *x*_*i*_, a *K* additive functions is used to predict the output of the tree ensemble model.

$$ {\hat{y}}_i=\phi \left({x}_i\right)=\sum \limits_{k=1}^K{f}_k\left({x}_i\right),\kern2em {f}_k\epsilon\ F $$

where *F* is the set of all possible trees. The *f*_*k*_ function maps the value in *x*_*i*_ to a certain output, at each step *k*.

Extreme Gradient Boosting tries to minimize the regularized object as follow:

$$ \mathcal{L}\left(\phi \right)=\sum \limits_il\left({\hat{y}}_i,{y}_i\right)+\sum \limits_k\Omega \left({f}_k\right) $$

where $$ \kern1em \Omega (f)=\gamma T+\frac{1}{2}\lambda {\left\Vert \omega \right\Vert}^2 $$.

In the above equation, *l* is a differentiable complex loss function (Mean Square Error). while the second term penalizes the complexity of the model in terms of number of leaves in tree *T* and vector of scores on leaves *ω* to avoid overfitting.

In general, boosting procedure outperforms the random forests. *Extreme Gradient Boosting* in [10] is an efficient and scalable implementation of the proposed Gradient Boosting framework by Friedman [14].

### Extremely randomized trees

The Extremely Randomized Trees is a tree-based ensemble method for supervised classification and regression problems. It involves on the randomization of both attributes and cut-points choices during the splitting of a tree node. In the extreme case, trees are totally randomized and the structures of them are independent of the learning sample output values. The strength of the randomization can be tuned to problem specifics by the appropriate choice of a parameter [15].

With respect to random forests, the Extremely Randomized Trees idea is to drop the use of the learning sample bootstrap copies, and instead of trying to find an optimal cut-point for each one of the K randomly chosen features at each node, it selects a cut-point at random [15]. From a statistical point of view, the idea to drop the bootstrap copies leads to an advantage in terms of bias because the cut-point randomization has an excellent variance reduction effect.

### Super learner

In this case, the multi-class problem can be divided into a binary classification problems collection: considering 4 classes (*C*_1_, *C*_2_, *C*_3_ and *C*_4_), it is possible to adapt binary classifiers for *C*_1_vs *C*_2_ or *C*_3_ or *C*_4_, *C*_2_ vs *C*_1_ or *C*_3_ or *C*_4_, *C*_3_ vs *C*_1_ or *C*_2_ or *C*_4_ and *C*_4_ vs *C*_1_ or *C*_2_ or *C*_3_, and then combine the results to make a past classification on the highest probability estimate. Note that, the approach presents a certain flexibility for the classifier for the different categories. In general, it is not possible to know a priori which learner will produce the best performance for a given prediction problem [21, 28], and the relative performance of various learners depends on the true-data generating distribution. The idea is to apply a set of candidate learners to the observed data and choose the optimal learner for a given prediction problem by using a cross-validation risk approach. Thus, the learner algorithm with the minimal cross-validation risk is selected. *Super Learner* performs asymptotically to produce the best possible weighted combinations among the set of candidate learners considered [26, 27].

Therefore, considering *L*(*n*) a collection of learners ${\hat{\Psi}}_l,\kern0.5em l=1\dots L(n)$, in parameter space **Ψ.**

*Super Learner* is defined as

$$ \hat{\Psi}\left({P}_n\right)\equiv {\hat{K}}_{\hat{L}\left({P}_n\right)}\left({P}_n\right), $$

where $\hat{K}\left({P}_n\right)$indicate the cross-validation selector.

$\hat{K}\left({P}_n\right)$selects the best performing learner in term of cross-validated risk:

$$ \hat{L}\left({P}_n\right)\equiv \underset{l}{\arg \min }{E}_{V_n}\sum \limits_{i,{V}_n(i)=1}{\left({Y}_i-{\hat{\Psi}}_l\left({P}_{n,{V}_n}^0\right)\left({X}_i\right)\right)}^2, $$

In particular, *V*_*n*_ ∈ {0, 1}^*n*^ indicates a random binary to split the learning data set into a training set {*i* : *V*_*n*_(*i*) = 0} and validation set {*i* : *V*_*n*_(*i*) = 1}. The empirical probability distributions of the validation sample and training sample are denoted by ${P}_{n,{V}_n}^1$ and ${P}_{n,{V}_n}^0$ respectively [28]. In the performed experiments, the *Super Learner* has been applied with 10-fold cross-validation to select the optimal learner given the following set of candidates: *Random Forest, Gradient Boosting, Extremely Randomized Trees*.

## Data and features definitions

The features considered by the classification algorithms have been selected from a larger set considered during the preliminary analysis and experiments. The process of features reduction has been performed by assessing their relevance in the contexts of the algorithms tested as mentioned in Section 3. The aim was to identify the smallest subset of features without reducing significantly the precision of the classifications. As a result, Table 2 includes the selected features7Metrics, classified in 4 categories, collected from the mobile as *Sensor Data Package*, with those computed from the server-side to be used by the classification algorithm. Some of these features can be used for both users’ transportation mean classification, and for creating firing conditions for implementing strategies. In Table 2, column “Where” is: “M” when the measure is produced on the Mobile Phone, and “S” when is computed on server-side.

**Table 2:** Overview of Sensor Data Package feature measured at a given time from the mobile or computed on server-side

Categories	Metrics	Description of metric variable	Where
Day/Time Baseline and GPS	Day and Time	Day and Time of the sample package	M
	Non-Working day	1 if weekend or vacation, 0 if it is a working day	M/S
	Time Slot	Slot of the day (morning, afternoon, evening, night)	S
	GPS latitude and longitude	Position of the mobile phone in GPS coordinates	M
	Accuracy	GPS Sensor’s Accuracy from the mobile phone	M
	Location Measure kind	Types of Location measure: GPS, Network, Mixed/Fused	M
	Speed	Speed as provided by the GPS driver of the mobile (as m/s)	M/S
	Average Speed	Average speed of the measures collected in the last two minutes	M/S
	Phone Year	Year/age of the terminal	M
	BDS	Availability of a BDS compliant GPS Sensor	M
	User Type	User Type: commuter, citizen, students, tourist, etc.	M/S
Accelerometer	Average linear magnitude of acceleration	Average of the acceleration magnitude calculate on five measurements	M
	Linear acceleration of X-axis	Acceleration of the mobile phone along the X-axis, purged by Earth gravity	M
	Linear acceleration of Y-axis	Acceleration of the terminal along the Y-axis, purged by Earth gravity	M
	Linear acceleration of Z-axis	Acceleration of the terminal along the Z-axis, purged by Earth gravity	M
Proximity	Rail Line	Bool indicating if the mobile phone is in proximity of a rail line	S
	Sport Facilities	Bool indicating if the mobile phone is in proximity of a sport facilities	S
	Tourist Trail	Bool indicating if the mobile phone is in proximity of a tourist trail	S
	Green Areas	Bool indicating if the mobile phone is in proximity of a green areas	S
	Bus/Light-rail Line	Bool indicating if the mobile phone is in proximity of a bus line or a light-trail line	S
	Cycle Paths	Bool indicating if the mobile phone is in proximity of a cycle path	S
Temporal window	Previous speed	Speed of the mobile phone of the previous 12 min	S
	Previous average speed	Average speed on the measures collected in a 12 min time slot	S
	Previous median speed	Median speed on the measures collected in a 12 min time slot	S
	Speed distance	Speed (m/s) calculated on the distance between two consecutive coordinates and the time passed between the observations	S

Each measure is collected/referred at a given *Day and Time.* Then it can be easily derived on the mobile or on server if the day is a working day or not (*Non-Working Day*). The same approach can be followed to detect the *Time Slot* in which the measure has been collected. The Time Slot strongly influences the attitude of the city users to move by using different transportation means.

As described in Section 2, the information about the user’s movements is collected from the mobile phone sensors. If the user has the mobile application in foreground, the data are sent to the server every 1 min and 30 s (sending interval). This interval can be reduced by the user (via the setting of the App) to request an update up to 30 s, to have a more accurate assistance. If the App is not used, the data collection is performed in background modality, thus the measures and sending rates may become up to 3/5 min, forced by the operating system/mobile-phone, which in some cases can hibernate the App. Therefore, in order to make the solution viable in real conditions (differently from the state-of-the-art solutions), a set of strategies and robust classification algorithms have been put in place. Among them, solutions for filtering noise and GPS errors, and for smoothing the sequence of the user locations (user trajectory) have been used.

A *Sensor Data Package l*_*i*_ represents the user context at a specific time *t*_*i*_ and is composed by the *GPS latitude and longitude* (according to a Location Measure kind), speed, and accuracy of the measure plus a list of N additional features (*feat-1…feat-n*):

$$ \left\{{l}_i={latitude}_i,{longitude}_i,{speed}_i,{accuracy}_i, feat-{1}_i,\dots, \kern0.5em feat-{n}_i\right\} $$

A user trajectory *t*_*ir*_ is a sequence of *l*_*i*_ that describes the movements of a user to move from *l*_*i*_ to *l*_*r*_*:*

$$ {t}_{ir}=\left\{{l}_i,\dots, {l}_r\right\} $$

A segment *s*_*uv*_ is a trajectory *t*_*uv*_ in *t*_*ir*_where a user keeps the same mobility mean:


The distance between *l*_*u*_ and *l*_*v*_ can be approximated by using flat-surface formulae between the two coordinates <*latitude*_*u*_*, longitude*_*u*_*>* and <*latitude*_*v*_*, longitude*_*v*_*>*.

**Fig. 5:**
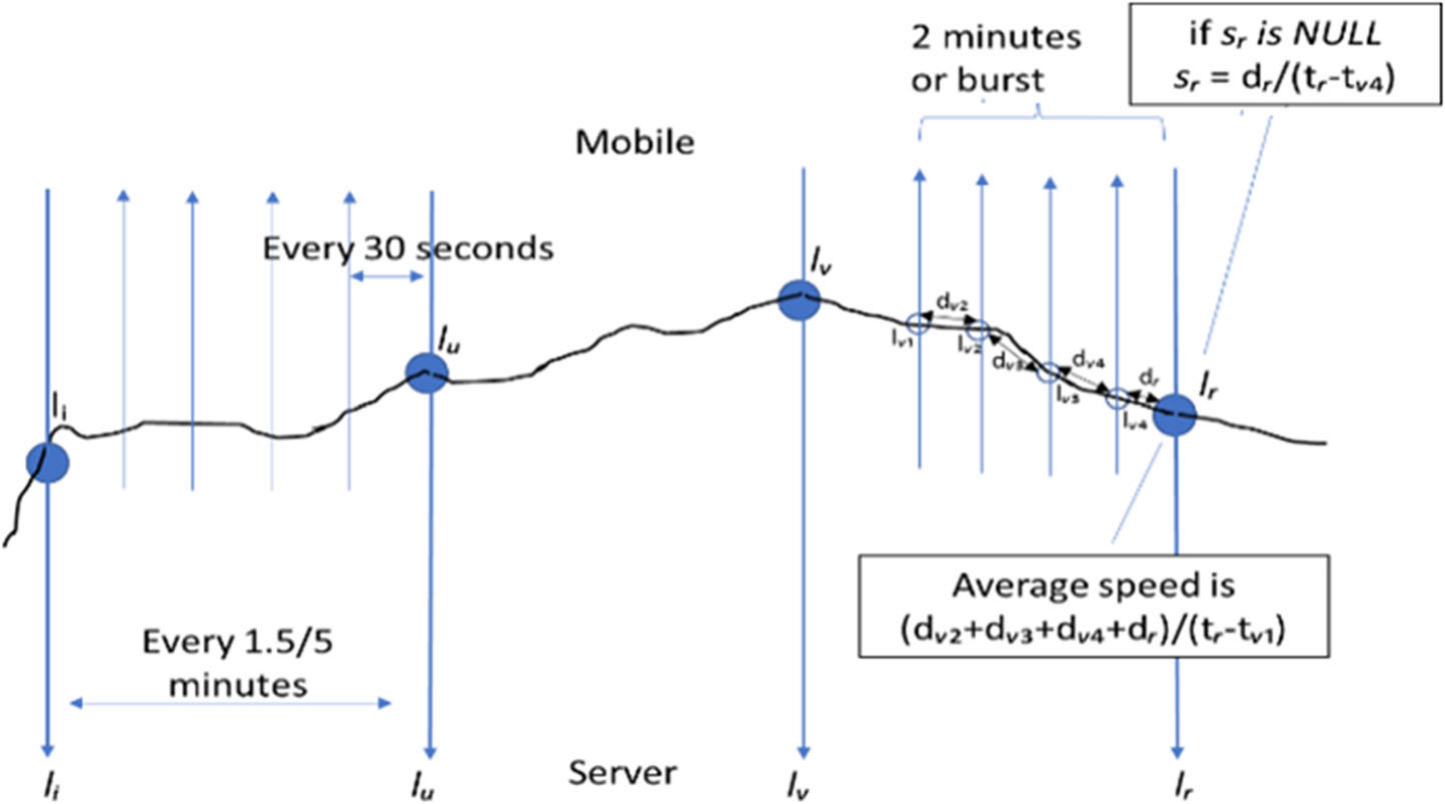
Speed and average speed

A measure of the mobile phone *Speed* can be directly retrieved from the GPS sensor (for example, every 30 s or at the rate imposed by the mobile phone). On the other hand, the above-mentioned Average Speed of Table 2 is calculated over the sequence of *l*_*i*_ in the same sending slot from the mobile phone, to cut out eventual errors coming from GPS sensor. If the mobile App is in foreground the *Average Speed* is computed every 2 min (4 measures of 30s, if any). If the mobile App service for collecting data is in background, and 2 min passed before a measure is available (probably the operating system put the application in hibernate mode). The service on the mobile tries to wake up whenever it is possible (if the operating system on the mobile phone allows us to wake the service up), to retrieve a bounce of new *l*_*i*_ to calculate a more precise Average Speed. If at a given time, the measure of Speed is not available, a “valid” value may be obtained on the server-side by using the distance between the two last GPS coordinates and the current refresh time. Figure 5 overviews the scenario.

The *Location Measure kind* is an important feature to understand the location measures reliability. Usually, the measures obtained and marked as “GPS” by the mobile phone are quite accurate, even if they suffer time by time of well-known problem of shading (e.g., urban canyoning) or blocked (under the bridge) [20]. The location measures, labelled as “Network”, resume the position from the location of the available Wi-Fi hotspots or GSM/4G/5G in the mobile connection; while those marked as “Mixed” modality is obtained by the operating system by merging the previous strategies according to different algorithms that may depend on the operating system kind, sensor kind, etc. The Location Measure kind strongly depends on the factory settings of the mobile phone, that make very difficult to force a pre-determinate modality from the App. On the other hand, the approach permits the users to optimize the battery use and to have more precise positioning in critical cases, thus the switch among modalities is not under control of the App. The **Accuracy** of the GPS measure is reported in meters from the mobile phone and can be used from the classificatory algorithm to eventually discharge entries.

Mobile Phone model and its characteristics are also tracked and passed to the classification algorithm. Thus, the mobile **Phone Year** of production and the characteristics of the GPS sensors strongly influence the reliability of measure and thus have and have been considered as variable, differently from the state-of-the-art solutions. Old mobile phones usually support just A-GPS modality, meanwhile new ones’ support also GLONASS and BDS standards. We have been also capable to experiments on new GALILEO compatible sensors available on new Samsung models. As a result, there is evidence that the type of the sensor influences the accuracy of location retrieval [17].

### Accelerometer features

The values from the *Accelerometers* of the mobile phone are always available and are sampled. Using the linear acceleration of the mobile phone avoids taking measures influenced by mobile phone orientation (horizontal or vertical, in the hand or in the pocket). Not all the mobile phones provide this information (some of them just return the non-linear values, influenced by the gravitational acceleration, and orientation, thus needing a de-rotation). On the other hand, almost all the relatively new mobile phones already provide this aggregated value. Mobile *Phone Year* variable allows us to take this into account. Thus, the three measures of linear acceleration on three axes have been considered aggregating five consecutive acceleration measures for computing an average magnitude as:

$$ Average\ Linear\ Magnitude\ of\  Acc={\sum}_{k=1}^5\frac{\sqrt{acc_{x_k}^2+{acc}_{y_k}^2+{acc}_{z_k}^2}}{5} $$

### Distance features

On the server-side, the *Sensor Data Package* collected from the mobile phone via the App is enriched by computing and, in most cases, exploiting the Km4City knowledge base of the City via Smart City API. This allows to retrieve contextual information about the closeness of the mobile phone/user with respect to: Railway Line, Sport Facilities, Tourist Trail, Green Areas, Bus/Light-rail Line, and Cycle Paths. The closeness features are binary values that specify if the location is closer to those structures, in the range of 30mt. This derived information is very valuable for understanding some transportation means. For example, to be close to a Rail and/or Bus/Light-rail line for a number of points of a trip permits to infer bus/train modality (train, bus and light-rail run just in their closeness) with a very high probability. On the other hand, the closeness to a cycle path cannot directly infer that a user is using a bike because the user can be in its proximity by a car and with similar speed or a bike can run also away for the cycling path.

### Temporal window features

Besides having instantaneous measurements about mobile phone/user’s mobility, the speed values in the last 12 min time-frame is also computed on server-side, as well as the average and mean value between these measurements. This allows to reduce the noise overcoming disruptive mobility conditions mainly related to traffic congestion or temporary signal absence. This is also due to the fact that, the service for collecting data on the mobile phone runs on a real application (foreground/background) conforming to the policy of “energy saving” of the user to have shortage of data for up to 3/5 min. So that, in real conditions, it is very important to avoid battery drainage warning, that may stimulate the user to un-install the App from the mobile phone. In order to perform an addition refinement on speed measures, mean and median speed and distance between GPS coordinates are also computed.

The **User Type** specified by the user in the App during installation or setup permits contribute to the classifications and to the strategies. The User Types can be classified in: citizen, commuter, student, tourist, etc. We noticed that different profiles present a different approach in everyday mobility and, so on the transportation mode they normally use.

## Results from classification/prediction models

According to the above-described metrics and data, the challenge was to predict the transportation means, whether an individual is stationary, or is walking, or moving on a motorized private transport (car or motorbike) or using a public transport (tram, bus or train). This section presents two subsections; the first includes some descriptive notes on dataset, the second the results of the classification models.

### Data description

**Fig. 6:**
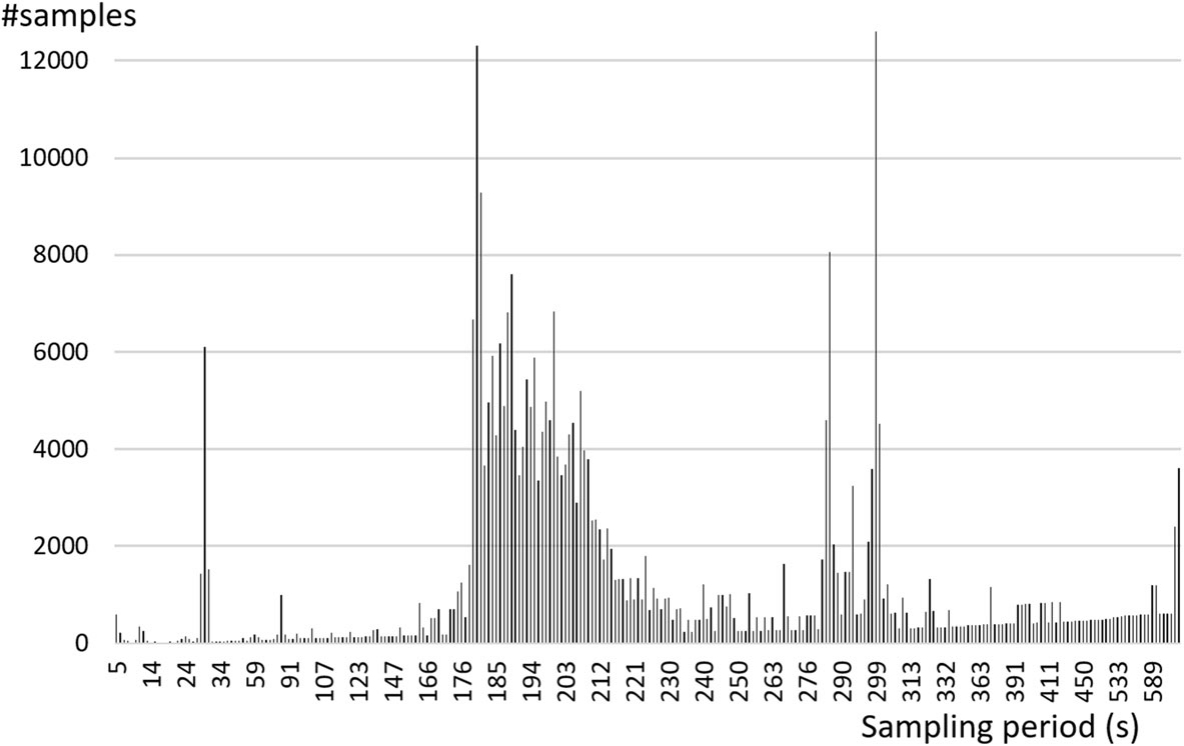
Distribution of Sampling Period/rate in s

The experiment has been conducted on about 30,000 observations, collected from April to August 2017 on 38 different users and 30 different kinds of mobile phones. Note that, each user can use the mean of transport they want. When the mode of transport is changed, the user was asked to notify the change to the App for creating the learning set and for validation. As mentioned above, no restriction was imposed on how the mobile phone should be held during movements (foreground/background, on hand or into bag, etc.). Unlike the experiments reported in the literature, most of the data was collected in the background because the phones were kept in the pocket or bag. In fact there is a non-conformity in the distribution of the sampling period of the collected data. In details, the period average is equal to 180 s and the variance is equal to 13,240 s. The distribution of the sampling period in seconds is reported in Fig. 6.

### Classification/prediction model results

The training set has been created by randomly selecting the 80% of the collected data, while the test set was the remaining 20%.

In the general framework, three different approaches were more successfully considered -- i.e., Random Forest (RF), Extremely Randomized Trees (Extra-Trees), and the Extreme Gradient Boosting procedure (XGBoost). Those approaches have been tested by using the above presented features/metrics (see Table 2), classified by categories as: *day/time baseline* and *GPS* features, *accelerometer* features, *distance* features and *temporal window* features. The comparison among those models has been reported in Table 3, in terms of resulting data. From the comparison, it is evident that all the approaches are capable to produce satisfactory predictions (the accuracy for each model exceeds 90%) for the identification of the transportation means.

**Table 3:** Classification Models Comparison on four classes of transport mode: stationary, non-motorized, private transport, public transport. Best values are  market in bold

Classifier Models	Accuracy %	Precision %	Recall %	F_1_ score
Extreme Gradient Boosting	94.7	77.3	82.8	0.800
Random Forest	94.2	77.4	86.9	0.819
Extra-Trees	**95.3**	**82.7**	**86.9**	**0.847**

Best values are market in bold

According to the data results of Table 3, the differences among the different approaches provide the evidence that the Extra-Trees resulted to be the better-ranked approach in terms of accuracy and *F*_1_score. In Table 3, the *F*_1_score is reported: *F*_1_score has been used to measure the models’ performances. This is a measure to evaluate the robustness of a model for making predictions, as a compromise between precision and recall. Moreover, we report here other definitions used in the following tables.

$$ {\displaystyle \begin{array}{c}\ {F}_1 score=2\times \frac{Precision\times Recall}{Precision+ Recall}\\ {} Precision=\frac{\#\mathrm{correctly}\ \mathrm{class}\mathrm{ified}\ \mathrm{istances}\ \mathrm{in}\mathrm{to}\ \mathrm{class}\ i}{\#\mathrm{istances}\ \mathrm{class}\mathrm{ified}\ \mathrm{as}\ \mathrm{class}\ i},\\ {}\begin{array}{c} Recall=\frac{\#\mathrm{correctly}\ \mathrm{class}\mathrm{ified}\ \mathrm{istances}\ \mathrm{in}\mathrm{to}\ \mathrm{class}\ i}{\#\mathrm{istances}\ \mathrm{belonging}\ \mathrm{to}\ \mathrm{the}\ \mathrm{class}\ i},\\ {} Specificity=\frac{\#\mathrm{correctly}\ \mathrm{not}\ \mathrm{class}\mathrm{ified}\ \mathrm{istances}\ \mathrm{in}\mathrm{to}\ \mathrm{class}\ i}{\#\mathrm{istances}\ \mathrm{not}\ \mathrm{belonging}\ \mathrm{to}\ \mathrm{class}\ i},\\ {}\begin{array}{c} Neg. Pred. Value=\frac{\#\mathrm{correctly}\ \mathrm{not}\ \mathrm{class}\mathrm{ified}\ \mathrm{istances}\ \mathrm{in}\mathrm{to}\ \mathrm{class}\ i}{\#\mathrm{istances}\ \mathrm{not}\ \mathrm{class}\mathrm{ified}\ \mathrm{in}\ \mathrm{class}\ i},\\ {} Balanced\ Accuracy=\frac{1}{2}\ \left( Recall+ Specificy\right),\\ {} Accuracy=\frac{\#\mathrm{correctly}\ \mathrm{class}\mathrm{ified}\ }{\#\mathrm{population}}\end{array}\end{array}\end{array}} $$

**According to this result**, the Extra-Trees algorithm achieves an accuracy of 95.3%, and a precision of 82.7%. It should be remarked that, these results have been obtained and can be produced by observing data coming from a large range of mobile phone kinds and a variable sampling rate (up to 5 min). The model produced allows to understand if a user is moving with a public or private transport.

On the contrary, in [24], a precision of 93.7% has been obtained by using a single mobile phone type, Nokia n95, and a constant sampling rate of 60s, which is not realistic with present mobile operating systems. With Reddy’s classification was only possible to know if a user is moving with a motorized vehicle. The same considerations apply to: [25] where data come from three different mobile phone types and they are taken with a constant rate of 15 s achieving a precision of 93.7%; and to [31] achieving a precision of 91% with accelerometer sensor data only, without distinguishing the type of motorized transport.

Moreover, Table 4 reports the assessment of the results performed for each traveling mean classification for the Extra Tree procedure according to our first result. The traveling mean class with lower accuracy is Walk. This is probably due to the fact that, it is not easily to understand if a user is walking or not, since the GPS sensors accuracy is very noisy in indoor scenarios, with frequent jumps passing from the different modalities: wifi-mixed, etc.

**Table 4:** Extra-Trees Prediction Model: Statistic by class

Extra Trees Model	Stay	Walk	Private Transport	Public Transport
Recall (Sensitivity)	97.8	73.1	86.9	91.7
Specificity	90.1	98.8	98.7	99.6
Precision	97.7	77.0	82.7	93.6
Neg. Pred. Value	90.4	98.5	99.0	99.4
Balanced Accuracy	94.0	85.9	92.8	95.6

### Combing with SuperLearner, second result

Subsequently, with the intention of improving the accuracy, the Super Learner algorithm has been applied by dividing the identified multi-class problem into four binary classification sub-problems, with 10-fold cross-validations, to estimate the risk on future data and select the optimal learner, on the basis of the above-mentioned set of candidates: Extra-Trees, RF, XGBoost. Then the results have been combined to make a classification on the highest probability estimate. Taking into account the classes of transport modality above (i.e., stationary, walking, private transport, public transport), four different binary classification models have been constructed:

(A) stationary vs walking, private transport, public transport.
(B) walking vs stationary, private transport, public transport.
(C) private transport vs stationary, walking, public transport.
(D) public transport vs stationary, walking, private transport.

The results for each binary classification model are reported in Tables 5. Where: RCV risk is a measure of model accuracy or performance (at lower value corresponds a lower risk and higher accuracy).

**Table 5:** Super Learner results on each binary Classification Model: Couples A to D

Method	RCV risk	*Coef*
(A) Stationary vs Walking, Private Transport, Public Transport.		
Extra-Trees	0.0282	0.5391
RF	0.0287	0.0562
XGBoost	0.0300	0.4047
(B) Walking vs Stationary, Private Transport, Public Transport.		
Extra-Trees	0.0234	0.6277
RF	0.0259	0.0091
XGBoost	0.0252	0.3632
(C) Public Transport vs Stationary, Walking, Private Transport.		
Extra-Trees	0.0213	0.6857
RF	0.0235	0.0000
XGBoost	0.0239	0.3143
(D) Private Transport vs Stationary, Walking, Public Transport.		
Extra-Trees	0.0087	0.6296
RF	0.0108	0.0000
XGBoost	0.0096	0.3704

The obtained results have been combined on the highest probability estimation. The complete statistic by class of Super Learner algorithm is reported in Table 6. Please note that, the coefficient columns indicate the weight of each individual learner in the overall ensemble, and the weight values are always greater than or equal to 0 and sum to 1.

**Table 6:** Binary Classification Models combination based on the highest probability estimate: Statistic by class

Super Learner Model	Stay	Walk	Private Transport	Public Transport
Recall (Sensitivity)	99.0	66.2	85.7	92.7
Specificity	89.2	99.3	99.0	99.6
Precision	97.5	83.1	86.5	95.3
Neg. Pred. Value	95.5	98.2	98.9	99.4
Balanced Accuracy	94.1	82.8	92.4	96.1

This is our second result. In this case, the average accuracy has been of 96%, precision of 86.5%, and a recall of 85.7%; with a *F*_1_*score* equal to 0.861. Therefore, the results obtained by using the Super Learner overcome those of the Extra-Trees multi-class model (see Table 3), compared in terms of average accuracy and F_1_score, and those from the literature. For each class of transportation modes, three different algorithms have been compared in terms of RCV risk. Please note, that Extra Trees has obtained the lower risk in all the binary classifications. Therefore, according to Tables 6 and 4, there are no significant differences in terms of Balanced Accuracy between the Super Learner approach and Extra Trees multi-class model, except for class *Walk* in which Extra Trees model is better ranked in terms of accuracy and sensitivity. *For this reason, the Extra-Trees model could still be the best choice.*

### Assessing the influence of features

A comparison in terms of accuracy, precision and recall of the Extra-Trees multi-class approach has been computed considering four combinations of the different categories of data (as reported in Table 2):

- baseline features and distance feature.
- baseline, distance feature and accelerometer features.
- baseline, distance feature and temporal window features.
- baseline, distance, accelerometer, temporal features together. (Full Model)

This set of combinations of feature categories permits to assess the flexibility of our approach in real operative conditions, where a variety of mobile phones have to be supported, since not all mobile phones support the full combination of data categories with the data they provide.

The results obtained by using different subsects of feature categories are reported in Table 7. Please note that the differences among the different cases for feature categories are substantial. The results suggest that the best choice in terms of precision is still the usage of model exploiting all the categories together, thus demonstrating that the model is flexible and resilient with respect to the mobile phone kind. Please note that the Boolean value detecting a close transportation line (i.e., proximity feature in the table) improves the classification effectiveness: the accuracy passed from 91% to 92% and higher.

**Table 7:** Extra Tree Model results on four classes of transport modality (stationary, non-motorized, private transport, public transport) considering four combinations of the different features

Model features categories	Extra Tree Model results			
	Accuracy %	Precision %	Recall %	F_1_Score
Baseline and GPS	91.0	68.2	75.1	0.714
Baseline and GPS + proximity	92.4	73.9	69.1	0.715
Baseline and GPS + proximity + Accelerometer	92.6	81.4	74.4	0.777
Baseline and GPS + proximity + Temporal window	94.9	80.5	78.7	0.787
Baseline and GPS + proximity + Accelerometer + Temporal window	**95.3**	**82.7**	**86.9**	**0.847**

Better ranked in the comparison are shown in bold

**Fig. 7:**
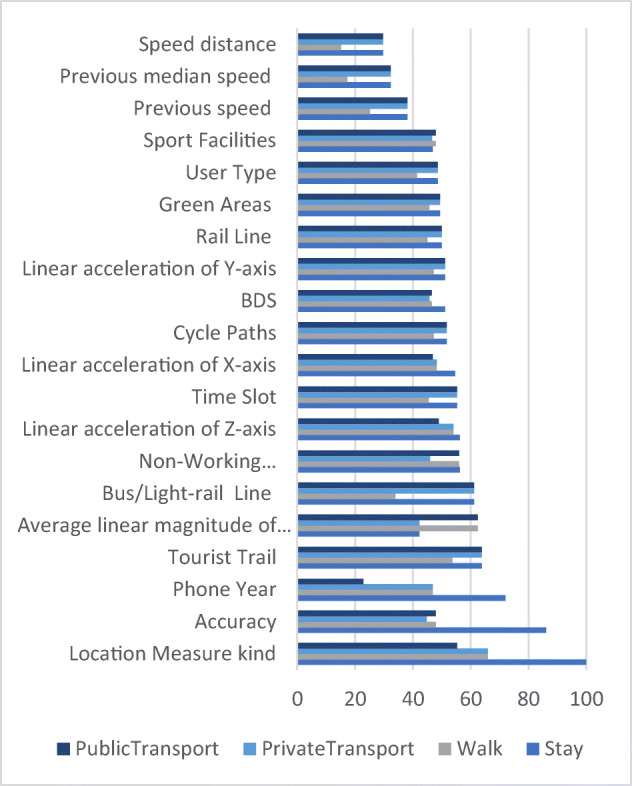
Variables Importance across the classes of the Extra-Trees full model

In Fig. 7, the features listed in Table 2 are reported in order of importance across the classes for the prediction of the Extra-Trees Full Model, (the model with all the categories of covariates). The distribution of relevance suggests that the variable Location Measure kind (i.e., GPS, Network or Fused/Mixed) is the most relevant for predicting the class of transportation mode, due to the fact that in Stay mode (during the night) usually the service is kept in background (thus the terminal operating system use a specific location provider to save battery usage).

The relevance of each predictor has been evaluated using the ROC curve analysis [11]. For multi-class outcomes, the problem has been decomposed into all pair-wise problems. The area under the curve has been calculated for each class pair (i.e., Stay vs Walk, Walk vs Private Transport etc.). The maximum area under the curve across the relevant pair-wise AUC’s is used as the variable importance measure of a specific class.

### Hierarchical approach

The above presented and adopted learning models reflect the one-step algorithm methodology. In this section, we compared those models with a two-step hierarchical approach in terms of final accuracy and testing execution time. The hierarchical approach can be considered a combination of the Extra-Tree multi-class classification and the Super learner algorithm. As first step, we can consider the Extra-Tree multi-class classifier (as reported in Table 3), from which the two transportation means are classified with higher probability. Subsequently, as second step, the Super Learner approach has been used to discriminate between these transportation means. A threshold has been used to decide which class can be considered directly correct at the first step: if the probability of the class is higher respect the considered threshold (0.90), the transportation modality is regarded correct without proceeding to the second step. In this case, it can be difficult to know a priori which machine learning method will work best [28], especially if the two transportation modes that have to be discriminated can variate among the different combination of transportation mode pairs: Super learner can be a solution to compare different approaches and find the best one or the best combination.

**Fig. 8:**
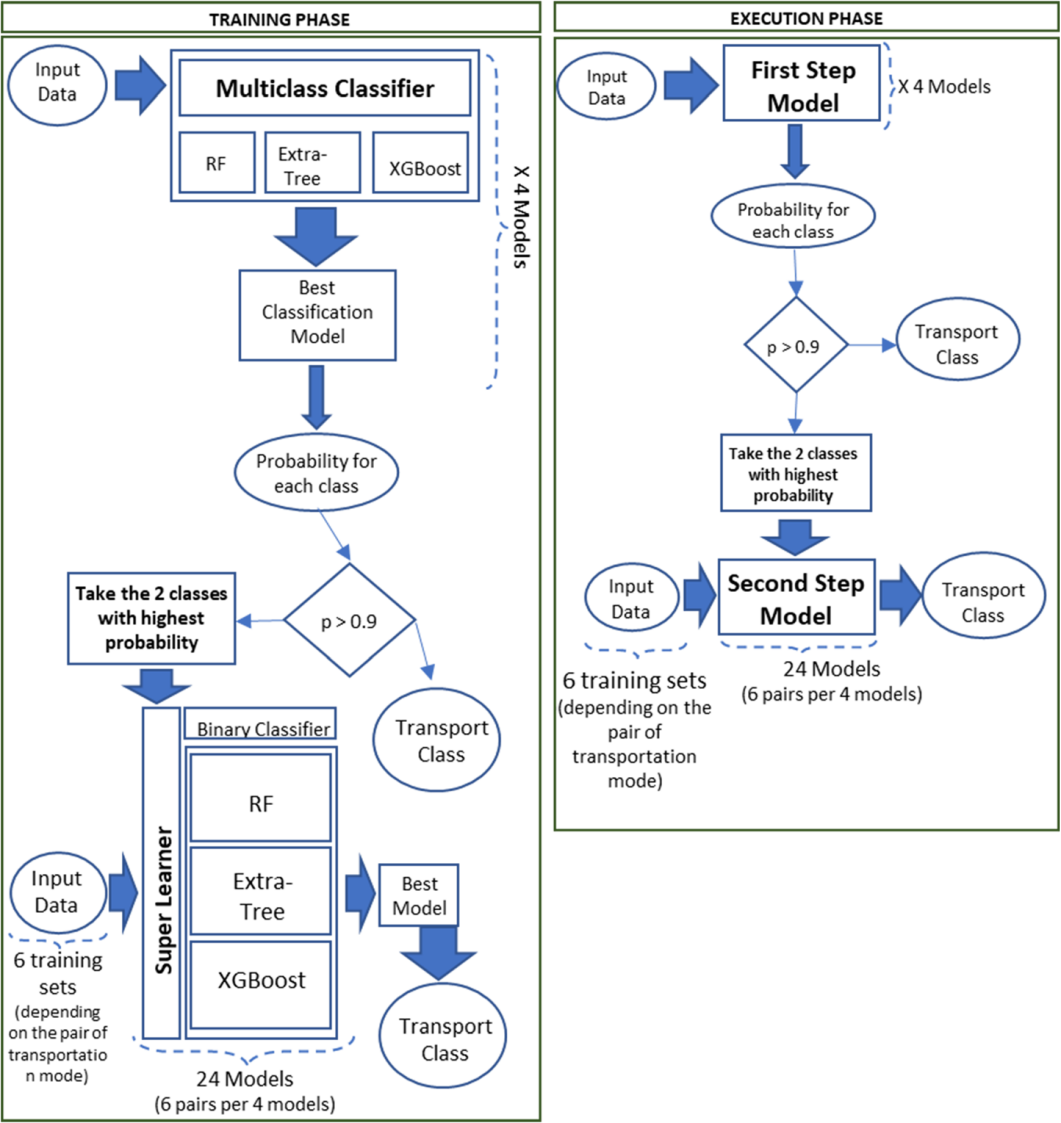
Scheme of the hierarchical approach in training and execution

The considered machine learning algorithms are the Extra-Tree, the Random Forest and the XGBoost. As reported in Fig. 8, the classes with higher probability respect to the threshold are not considered in the step two of the hierarchical model: the classification has been considered correct and no corrections have been made in the second step using the second learning approach (Super Learner).

In the first step, the Extra-Tree algorithm achieved a 97.6% precision for the classes with probability higher than the threshold. In the second step, six new binary classification models have been created, one for each combination between pairs of the transportation modes selected during the step-one (the classes with a probability lower than the threshold, i.e., Stay-Walk, Stay-Private Transport, Stay-Public Transport, Walk-Private Transport, Walk-Public Transport, Private Transport-Public Transport).

Table 8 reports the confusion matrix related to the hierarchical approach (Extra Tree – Super Learner). Note that, Super Learner algorithms takes a weighted average of the learners using the coefficients/weights, with 10-fold cross-validation. For the two steps hierarchical approach, average accuracy is 94%, while precision and recall are 78.6% and 86.9% respectively. It is also interesting to note that the average accuracy of each combination of transportation modality significantly decrease respect to the first step. This can be due to a loss of information from the first to the second step. In fact, after the application of step-one (Extra-Tree algorithm), for the classes with a probability below the threshold, the achieved accuracy is higher respect to the average accuracy calculated during the step-two (80.8% and 77% respectively). Moreover, the percentage of transportation modalities correctly classified during the step-one is of the 75%, while the remaining 25% of the test sample is further classified during the step-two.

**Table 8:** Two-steps hierarchical approach confusion matrix (Extra-Tree and Super Learner considering Baseline, GPS, proximity, Accelerometer and Temporal window features)

Two-Steps Hierarchical Approach		Predicted			
		Stay	Walk	Private Transport	Public Transport
Actual	Stay	0.98	0.30	0.09	0.03
	Walk	0.01	0.60	0.02	0.01
	Private Transport	0.01	0.07	0.87	0.07
	Public Transport	0.00	0.03	0.01	0.89

### Real condition scenario vs hierarchical approach limitations

Several considerations have been already presented about the critical aspect of working on real operating conditions.

Battery drainage and the opportunity to support a contextual service for the users, even with the application in background mode, drove our research, despite little decrease of accuracy and precision. We decided to design a client-server architecture to support a finer classification, using GIS data easier available on the server side (avoiding user terminal network bandwidth usage to eventually download from remote) and to support technologies to aggregate information cross-terminal and user agnostic. Implementing a central server-side classification algorithm leaves open also the chance to auto-update scenario with feedbacks provided directly by the user. However, a real condition scenario can be affected by some limitations that cannot be solved either if a hierarchical approach is applied. This is due to the fact that the phone/user characteristics can be manifold, e.g., the presence of accelerometer information, the different type/generation of GPS sensor, the presence of information related to the temporal window, etc. For this reason, the classification model has to be flexible and the training dataset has to be as much as possible various (e.g., any kind of generations, manufactures, years, characteristics, etc.) without any restriction. The application of a two-steps approach, as demonstrated in the previous subsection, may lead to a loss of accuracy due to a loss of information and can be more time consuming in terms of execution time and number of different training models. In detail, during the second step, six different training models have to be executed, one for each combination between pairs of the transportation modes (selected during the step-one), considering that the classes of transportation means are four. In addition, a specific model has to be created depending on the characteristics of the mobile phone and of the users, considering four combinations of the different categories of data (reported in Table 2). Therefore, 4 different training models during the first step, and 24 different training models during the second step (6 transportation modality pairs combinations per 4 categories combinations) have to be computed.

### Composed solution

In the previous subsections a comparison between different solutions has been presented and discussed. On one hand, a two-steps hierarchical approach has been proposed. In the first step a multi-class classifier algorithm has been adopted to classify the transportation modalities. After the first classification, the classes with a probability lower than a threshold of 0.90 (*prob* < 0.90) have been re-classified in the second step, while the classes that have a probability higher than 0.90 are considered as correct and excluded from the re-classification test set. During the second step six different binary classification model have been trained, one for each pair of transportation modality.

On the other hand, a single step classification model has been presented and different models have been compared. The Extra-Tree algorithm can be considered as the best solution: it was found to produce the best performance in terms of average accuracy (95.3%) and time consuming. In detail, four different models have been trained to make the approach as flexible as possible. The necessity of this flexibility is because the solution has to be applied in a real condition scenario, for different phone/user characteristics, in any pseudo real-time context.

The advantage of this solution is not only in terms of accuracy but also in terms of number of training models (4 different models vs 4 + 24 different models in the hierarchical solution). Therefore, the hierarchical classification cost in terms of time 30 times more than the average of the other classifiers.

### Results from the applicative scenario

The prediction model proposed has been created by exploiting open and real-time data of the Sii-Mobility (national smart city project of Italian Ministry of Research for terrestrial mobility and transport, http://www.sii-mobility.org). The solution presented has been deployed on Smart City Apps in the Tuscany and Florence areas for sustainable mobility, which is now in place for stimulating the private mover toward a more sustainable mobility with the collaboration of public transportation operators.

The User Engagement exploits the above presented classification model proposed. The validation performed has been based on more than 400,000 engagement messages on more than 10,000 users with around 62,000 daily users’ profiles analysed. During the Pilot period (from 15th of April to 15th July) 654 users registered in the system, and the Mobile App was download 1443 times. The budget for rewarding collected by the TPL was around 2000 euros that has been dispatched to a total of 93 users (48 for Pisa, 26 for Prato, 19 for Firenze). The promotion of the Pilot has been carried out via Facebook (3 posts on highlight), via email to registered users and via flyer and banner located directly on bus. The set of rules created to dispatch engagement messages was defined directly by the public transport operators with the aim of creating a change of user behaviour, from private travel means to public transport solutions.

**Fig. 9:**
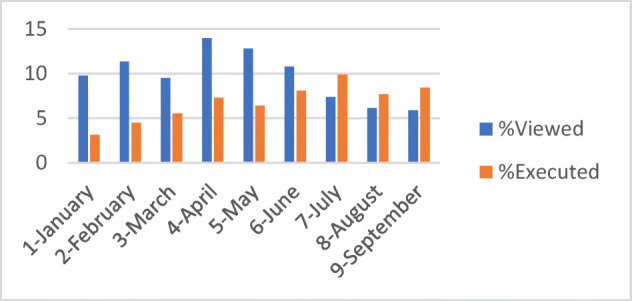
Trend of percentage of engagement messages viewed and accepted/executed by the users in the period, with respect to the total amount of personalized messages delivered

A detailed analysis on the level of proactivity in the use of the Mobile App was carried out. The analysis on each specific type of engagement message highlighted the contextualization of the message is also high influenced by the type of interaction required by the user. Figure 9 presents the percentages of the viewed messages that have provoked engaged actions by the users, the evidence of the incremented engagement is provided.

## Conclusions

This research has been focused on presenting a solution to create a classification system that uses mobile phone devices’ sensor values and GIS data (user contextual information) to identify the transportation mean of users: stationary, walking, on a motorized private transport (car or motorbike) or in a public transport (tram, bus or train). The goal has been to define a solution for sustainable mobility, delivering to the user useful personalized assistance messages. A number of metrics and features have been chosen as the *baseline and GPS*, the *distance*, the *accelerometer* data and the *temporal windows* data. The research documented in this paper demonstrated that a one-step multi-class classifier solution was found to produce the best performance in terms of average accuracy and time consuming if compared to a hierarchical approach. In detail, the Extremely Randomized Trees exploiting all the discussed above data can be a robust approach for reliable, precise and fast estimation of transportation means. The proposed solution overcome those of the literature since it presents a solution that is capable to produce reliable results in real conditions (i.e., real-time applications and background modality of operations) with a real set of mobile phone kinds and in particular: (i) addressing a large number of mobile phone kinds providing different features, different GPS sensors, different accelerometer sensors, etc., (ii) working with time variable samples of the data that may be due to the different operating systems, energy saving setting, etc., which are not under control of the App and thus are a strong constraint to realize real applications, background/foreground modality of operation; (iii) exploiting a number of different features and obtaining results with higher precision and accuracy. For these reasons, features related to the type of phone, e.g., the presence of accelerometer, phone year, location provider etc., have been considered in the prediction model, contributing to perform corrections in the model.

The solution presented has been deployed on Smart City Apps in the Tuscany and Florence areas for sustainable mobility for stimulating a sustainable mobility in collaboration of three major operators: ATAF, BUSITALIA and CTTNORD. Recently the solution is used in Snap4City mobile Apps in Antwerp and Helsinki.
